# Impact of policy shifts and festival holidays on tuberculosis surveillance: a time-series analysis in Southwest China (2014–2024)

**DOI:** 10.3389/fpubh.2026.1900071

**Published:** 2026-09-23

**Authors:** Qinwen Luo, Qin Gong, Yan Zheng, Danrong Yang, Shicui Wang

**Affiliations:** Department of Tuberculosis Prevention and Control, Center for Disease Control and Prevention of Jiulongpo District, Chongqing, China

**Keywords:** Active Case Finding, epidemiological standardization, holiday effect, model benchmarking, pulmonary tuberculosis, time-series analysis

## Abstract

**Background:**

Accurate forecasting of pulmonary tuberculosis (PTB) is frequently impeded by surveillance artifacts, most notably health policy shifts and festival holidays. This study proposes a practical epidemiological forecasting framework to address these real-world disruptions within China’s monitoring data.

**Methods:**

We utilized an 11-year historical cohort (2014–2024) from Jiulongpo District, Chongqing. To ensure longitudinal comparability, we first performed an epidemiological standardization to mitigate the artificial structural break caused by the 2018 diagnostic reclassification. To establish a robust “biological baseline” (the natural transmission trend devoid of reporting artifacts), a parsimonious SARIMA model was benchmarked against alternative statistical and modern machine learning algorithms, and validated via a multi-year rolling window cross-validation (2022–2024).

**Results:**

Rolling cross-validation confirmed SARIMA’s temporal stability, effectively avoiding the overfitting tendencies observed in complex machine learning models. The optimal SARIMA(0,1,1) × (0,1,1)_12_ model demonstrated high predictive accuracy for the 2024 annual caseload (Relative Error: 6.17%). Notably, actual incidence in January and February fell significantly below the forecasted 95% prediction intervals (PIs), which was immediately followed by a reported “catch-up” spike in March. This reflects a temporary reporting disruption associated with the Lunar New Year holidays, acting as a socio-cultural filter.

**Conclusion:**

Epidemiological standardization is an important prerequisite for accurate trend evaluation amidst policy shifts. Furthermore, by treating holiday-associated deviations as diagnostic indicators, this rigorously benchmarked framework functions as an anomaly detection tool to estimate potential notification delays. Although broader geographic application requires localized recalibration, this strategy provides crucial evidence for post-holiday targeted Active Case Finding (ACF).

## 1 Introduction

Tuberculosis (TB) continues to pose a significant global public health challenge. Despite being both preventable and treatable, it remains a leading cause of death from a single infectious agent. China bears a considerable disease burden, ranking fourth worldwide in terms of incident cases (1). Although the national incidence has exhibited a gradual decline, notable regional disparities remain, particularly in western urban centers such as Chongqing. As a typical high-density megacity experiencing rapid urbanization, its high population mobility poses continuous challenges to localized TB control efforts (2–4). Consequently, accurate forecasting of TB incidence is essential for health authorities to optimize resource allocation and implement timely interventions.

Time series forecasting has emerged as a vital tool in epidemiological surveillance. For respiratory infectious diseases like pulmonary tuberculosis (PTB), which display pronounced annual periodicity (5, 6), the Seasonal Autoregressive Integrated Moving Average (SARIMA) model presents a distinct theoretical advantage by effectively capturing cyclical fluctuations driven by climatic and social factors (7–9). Recent advancements demonstrate the crucial value of predictive modeling in supporting public health surveillance and resource allocation (10). Consequently, modern epidemiology increasingly integrates conventional time-series baselines with collaborative machine learning frameworks to maintain operational stability across complex landscapes (11). However, the application of predictive models in real-world public health surveillance is frequently compromised by systemic biases and surveillance artifacts.

In China, two major artifacts significantly distort historical TB data. First, policy-induced structural breaks. The implementation of the new Classification of Tuberculosis (WS 196–2017) standard mandated the inclusion of tuberculous pleurisy in PTB reporting (12). This policy shift artificially inflated case numbers, rendering raw historical time series unsuitable for direct modeling. Most existing studies either overlook this structural break or discard historical data entirely, resulting in biased predictions or truncated training sets. While advanced analytical techniques—such as interrupted time-series (ITS) analysis (13), Bayesian structural time-series (14), or change-point detection—are robust for evaluating the causal impact of such interventions, our primary objective is operational forecasting rather than causal inference. Second, sociocultural disruptions, notably the “Spring Festival Effect”. Characterized by substantial population migration and a traditional reluctance to seek healthcare during the Lunar New Year, this festival introduces massive behavioral noise into monthly reporting data, masking the true transmission dynamics (15, 16).

To bridge these methodological gaps, this study employs Jiulongpo District—a core urban area in Southwest China—as a representative case study to propose a practical epidemiological forecasting framework. Our specific objectives are: (1) to standardize an 11-year historical cohort (2014–2024) by retrospectively harmonizing data to mitigate the 2017 policy-induced structural break; (2) to build and benchmark a parsimonious SARIMA model against a suite of alternative approaches (including standard ARIMA, exponential smoothing, and machine learning algorithms) to establish a transparent “biological baseline” (representing the natural transmission trend) of TB transmission dynamics; (3) to evaluate model generalizability and temporal stability using a multi-year rolling window validation scheme (2022–2024); and (4) to utilize this calibrated baseline as an anomaly detection tool to quantify the “hidden” backlog of un-reported cases caused by the Spring Festival, thereby providing scientific evidence for targeted active screening and resilient post-holiday TB control strategies.

## 2 Methods

### 2.1 Data collection and epidemiological standardization

Monthly data on reported cases of pulmonary tuberculosis (PTB) in Jiulongpo District, Chongqing, were obtained from the “China Disease Control and Prevention Information System” (CISDCP). The study period spanned from January 1, 2014, to December 31, 2024. Data from 2025 onwards were excluded because a local adjustment in public health jurisdiction altered the population denominator, rendering subsequent surveillance data epidemiologically incomparable with the historical cohort. To establish a robust baseline for time-series forecasting, it was necessary to standardize the diagnostic criteria across the study period. In May 2018, China implemented the new Classification of Tuberculosis (WS 196–2017) standard, which mandated the inclusion of tuberculous pleurisy into total PTB counts (12). Prior to this policy shift, tuberculous pleurisy was recorded as a separate notifiable infectious disease. To eliminate the artificial structural break caused by this administrative change, we retrospectively extracted independent records of tuberculous pleurisy from 2014 to 2018 and aggregated them into the main PTB dataset. This essential data harmonization step ensured a unified and consistent definition of PTB cases throughout the observation window. For primary baseline modeling, the harmonized dataset was divided into a training set (January 2014 to December 2023, 120 months) and an out-of-sample validation testing set (January to December 2024, 12 months). In this study, we utilized absolute case counts rather than population-standardized incidence rates. While standardized rates control for demographic shifts, high-frequency (monthly) district-level population denominators are subject to significant reporting lags and high mobility fluctuations in Chinese megacities. Furthermore, for local health authorities, absolute disease burden is the primary operational metric for immediate resource allocation (e.g., hospital beds, staffing, testing kits) during post-holiday periods (17).

### 2.2 Descriptive analysis and time series decomposition

Descriptive statistics were employed to characterize the baseline demographic variables, including sex, age, and occupation, as well as the spatial distribution of cases. The temporal trend of annual incidence was assessed using the Cochran-Armitage trend test (18, 19). To identify the underlying components of the time series prior to modeling, we applied the Seasonal-Trend decomposition procedure based on Loess (STL) (20). This method decomposed the original time series into trend, seasonality, and remainder components, which subsequently informed the selection of differencing orders for the SARIMA model.

### 2.3 SARIMA model construction

The Seasonal Autoregressive Integrated Moving Average (SARIMA) model was utilized to capture both the trend and seasonal characteristics of the time series. The model is expressed as *SARIMA*(*p*,*d*,*q*) × (*P*, D, Q)*_s_*, where (*p*,*d*,*q*) denotes the non-seasonal order, (*P*, D, Q) indicates the seasonal order, and *s* represents the seasonal period, which is set to *s* = 12 for monthly data.

The modeling process commenced with an assessment of stationarity. A Box-Cox transformation was applied to stabilize the variance (21), and stationarity was achieved through the necessary non-seasonal (*d*) and seasonal (*D*) differencing, as confirmed by the Augmented Dickey-Fuller (ADF) test (22) and visual inspection of the Autocorrelation (ACF) and Partial Autocorrelation (PACF) plots. For model identification, we employed a hybrid strategy to ascertain the optimal structure. Initially, an automated algorithm suggested a baseline model, which was followed by the manual construction of candidate models, including the classic “Airline Model,” to strictly enforce seasonal differencing based on the results of the STL analysis. The candidate models were subsequently compared using the Akaike Information Criterion (AIC) (23) and the Bayesian Information Criterion (BIC) (24). In adherence to the Principle of Parsimony, priority was given to the model with the lowest BIC value to mitigate the risk of overfitting. Finally, model adequacy was verified by examining the residuals using the Ljung-Box *Q* test (25) and ACF plots to ensure that they exhibited white noise characteristics.

### 2.4 Model benchmarking and multi-year rolling validation

To rigorously evaluate the predictive performance and stability of the proposed *SARIMA*(0,1,1) × (0,1,1)_12_ framework, two extensive validation schemes were implemented.

First, model benchmarking was conducted using the 2024 test set by evaluating the proposed model against three representative baseline families: (1) classical time-series architectures, represented by a standard *ARIMA* (1,1,1) without seasonal components; (2) exponential smoothing methods, specifically Holt-Winters Additive Exponential Smoothing; and (3) machine learning algorithms, represented by Random Forest Regression trained on multi-lag autoregressive feature sets (*Lag*_1_ to *Lag*_12_). Additional architectures, including Facebook Prophet and XGBoost Regression, were evaluated in preliminary sensitivity analyses and are fully documented in Supplementary material. To ensure full reproducibility, comprehensive details regarding feature engineering, software implementation, and the specific hyperparameter settings for all benchmark models are provided in Supplementary Text S1.

Second, a multi-year rolling window cross-validation scheme was performed across 2022, 2023, and 2024 to evaluate model stability against temporal fluctuations and avoid single-split bias. For each evaluation year *t*∈in {2022, 2023, 2024}, the model was trained on all available historical data prior to January of year *t* and forecasted over the subsequent 12-month horizon of year *t*.

### 2.5 Forecasting metrics and statistical analysis

The optimal model was employed to predict the monthly incidence for 2024. Predictive accuracy was evaluated by comparing the forecasted values with actual surveillance data, utilizing the Root Mean Square Error (RMSE), Mean Absolute Percentage Error (MAPE), and Relative Error. Additionally, 95% prediction intervals (PIs) were generated for all point forecasts to explicitly quantify forecasting uncertainty and expected variance. A two-sided *p*-value of less than 0.05 was deemed statistically significant. Data management was conducted using Microsoft Excel, while all statistical analyses were performed with R software version 4.3.1 (R Foundation for Statistical Computing, Vienna, Austria). The time series modeling and forecasting were specifically executed using the forecast and tseries packages (26).

## 3 Result

### 3.1 Demographic and spatial baseline characteristics

From 2014 to 2023, the study encompassed 6,527 confirmed PTB cases. The demographic composition showed a male predominance (67.41%) with a significant prevalence among the older adults (≥65 years, 20.12%) and the unemployed (38.07%) (Supplementary Table S1). Geographically, the outbreak displayed distinct clustering in densely populated commercial and transportation centers. The primary five subdistricts and towns, notably Yangjiaping and Shiqiaopu, represented more than 50% of the total cumulative cases (Supplementary Table S2). These consistent demographic and spatial trends establish the foundational epidemiological context for subsequent time series modeling.

### 3.2 Time series decomposition and trend analysis

The retrospective harmonization of the 2014–2023 dataset revealed a non-stationary time series characterized by distinct seasonal and trend components, necessitating pre-processing for SARIMA modeling. Although the overall annual incidence exhibited a statistically significant declining trend (*p* < 0.001) according to the Cochran-Armitage test, the temporal evolution displayed a distinct level shift during the 2017–2018 period (Supplementary Table S3; Figure 1). This structural break coincided precisely with the external policy intervention associated with the implementation of the WS 196–2017 standard, thereby confirming its direct impact on surveillance data and underscoring the necessity for the retrospective data harmonization conducted in this study. As empirically demonstrated in Supplementary Figure S1, this retrospective aggregation provides strong empirical support that the adjustment effectively smoothed out the artificial incidence surge triggered in May 2018. Importantly, it strictly preserves the natural biological seasonality of the original series, thereby yielding a more comparable historical baseline Consequently, this period marked a transition from a stable phase (2014–2016) to a peak induced by policy, which was subsequently followed by a gradual decline from 2019 to 2023. Crucially for model parameterisation, the STL decomposition and seasonal boxplots (Figure 2; Supplementary Figure S2) confirmed a robust annual periodicity (s = 12). A dominant seasonal peak was consistently observed in spring (March to May), contrasting with a distinct trough in winter (January and February). This pronounced seasonality, coupled with the underlying trend, underscores the necessity of both non-seasonal and seasonal differencing to achieve stationarity in the subsequent model construction.

**Figure 1:**
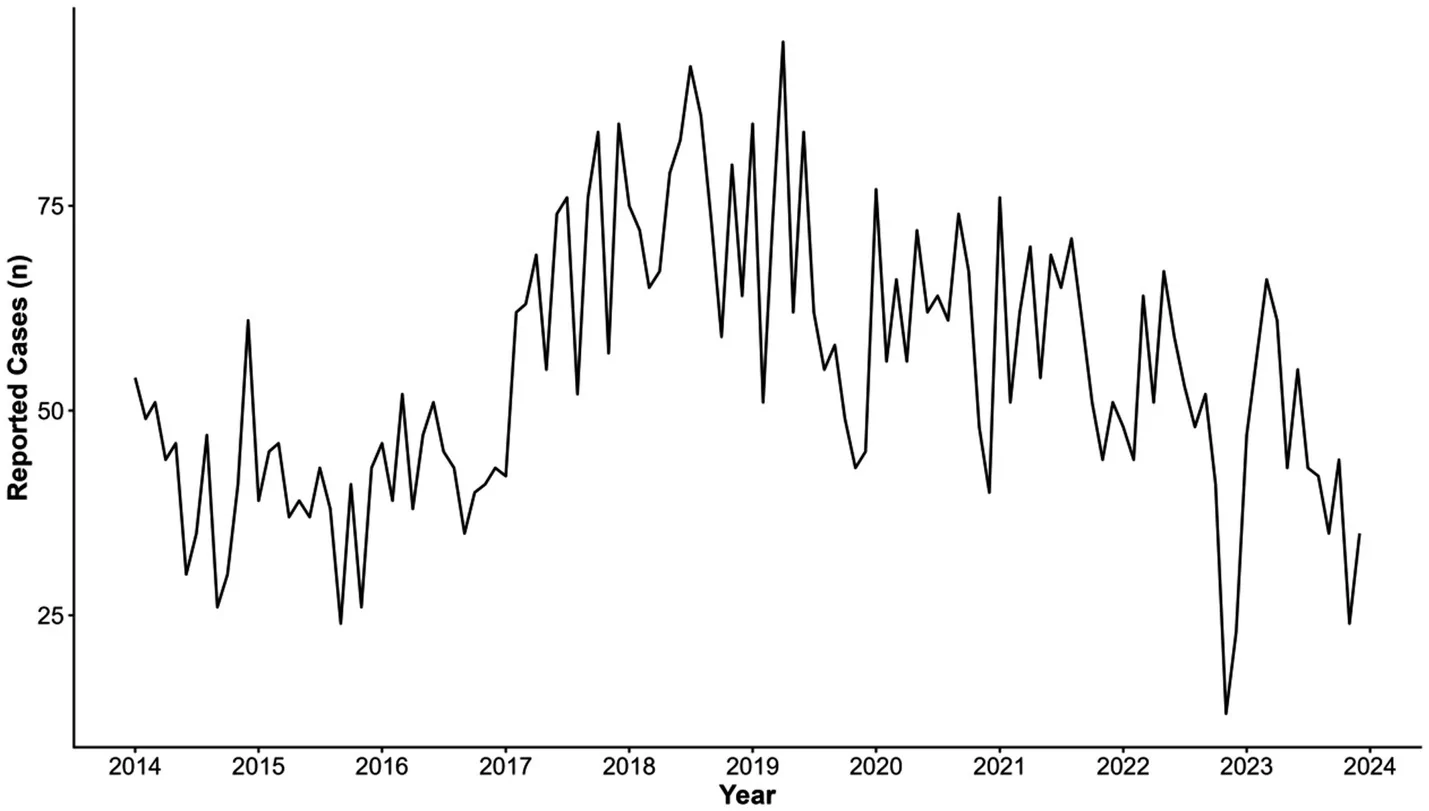
Time series distribution of pulmonary tuberculosis in Jiulongpo District, Chongqing, January 2014–December 2023.

**Figure 2:**
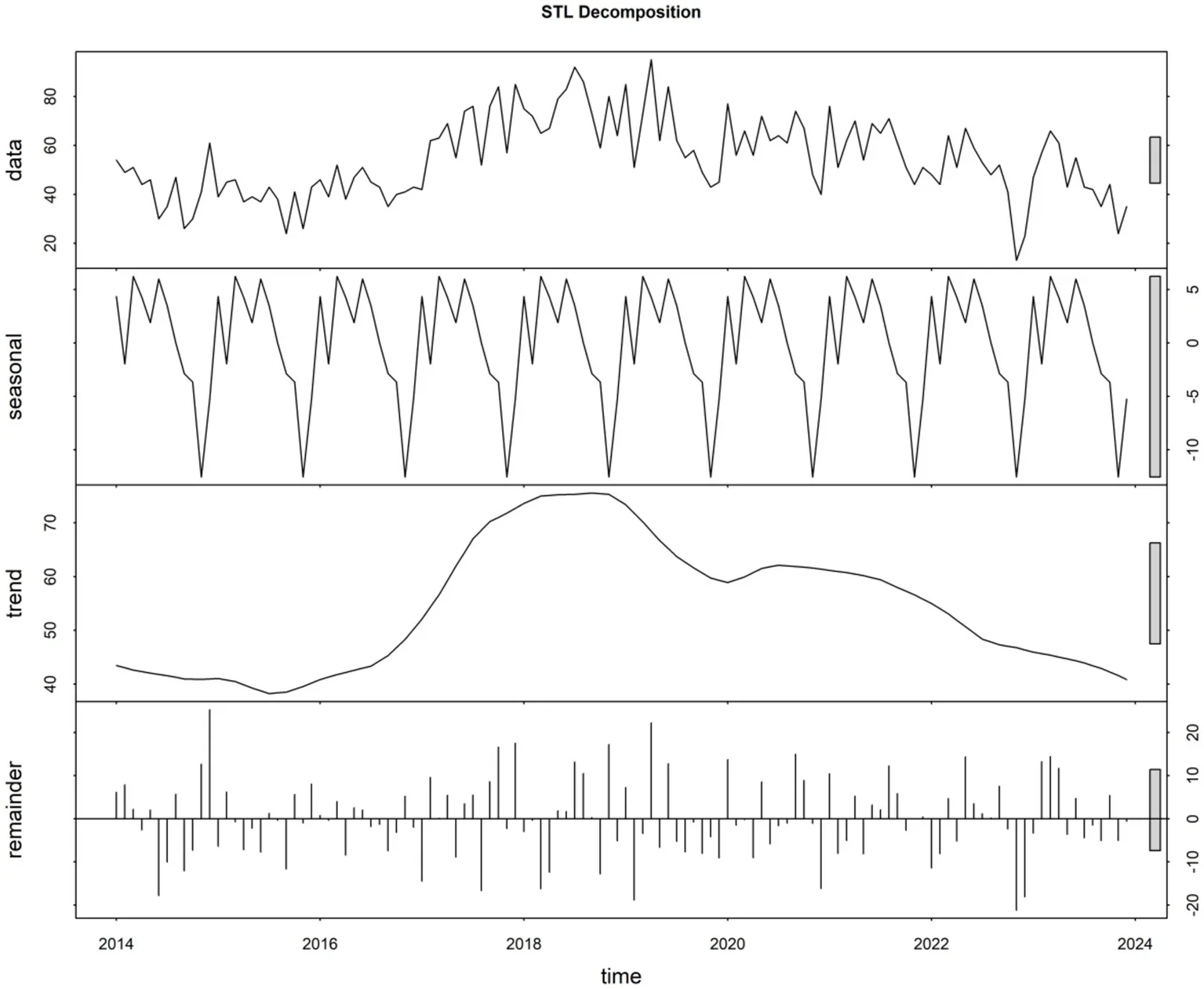
Trend, seasonal and residual components derived from STL decomposition of monthly reported pulmonary tuberculosis cases for Jiulongpo District, Chongqing, 2014–2023.

### 3.3 Model identification and selection

To stabilize the variance and eliminate the non-stationarity identified in the decomposition analysis, the original series underwent a Box-Cox transformation (*λ* = 0.50), followed by first-order non-seasonal (d = 1) and seasonal (D = 1, s = 12) differencing. The autocorrelation function (ACF) and partial autocorrelation function (PACF) plots of the differenced series (Figure 3) exhibited rapid decay within the 95% confidence intervals, confirming the achievement of stationarity. During the model selection phase, we employed a hybrid approach that combined automated algorithms with manual verification. The automated algorithm initially proposed a structure devoid of seasonal differencing (D = 0, AIC = 459.25), which resulted in inadequate goodness-of-fit. Consequently, we manually constructed six candidate models that incorporated seasonal components (Table 1). A critical comparison was conducted between the two top-performing models: the complex SARIMA(0,1,1) × (1,1,1)_12_ and the parsimonious SARIMA(0,1,1) × (0,1,1)_12_ (the classic “Airline Model”). Although the complex model yielded a marginally lower Akaike Information Criterion (AIC) (428.55 vs. 429.05), the difference was negligible (ΔAIC < 2). However, the Airline Model demonstrated a superior Bayesian Information Criterion (BIC = 437.06 vs. 439.24). In adherence to the Principle of Parsimony to prevent overfitting, we selected SARIMA(0,1,1) × (0,1,1)_12_ as the optimal model. This structure effectively captures the moving average dynamics of both the immediate past and the seasonal cycle while minimizing model complexity.

**Figure 3:**
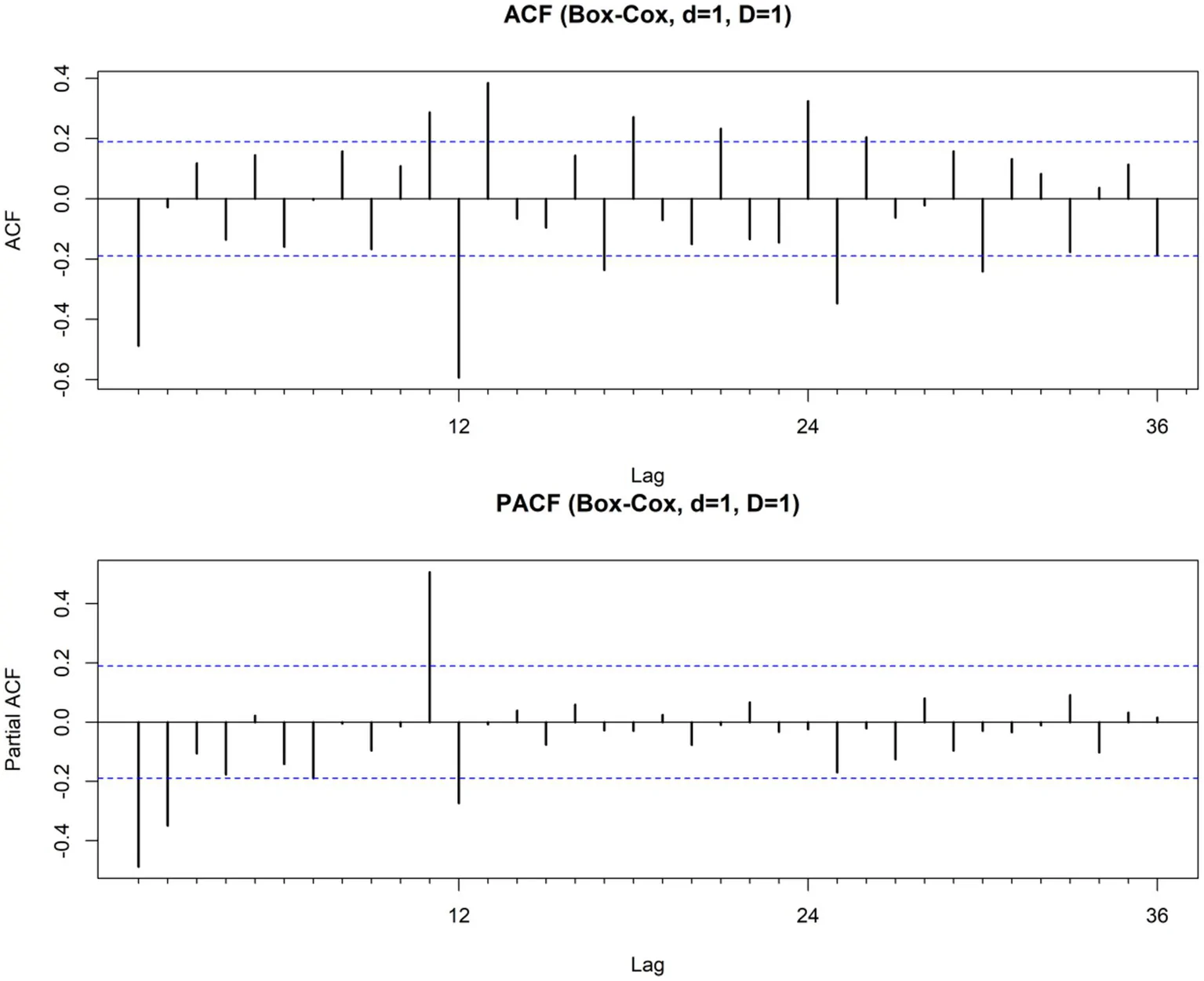
Autocorrelation (ACF) and partial ACF charts of monthly pulmonary tuberculosis cases.

**Table 1:** Comparison of goodness-of-fit statistics for candidate SARIMA models.

Model	AIC	BIC	RMSE	MAPE (%)
SARIMA(0,1,1)(1,1,1)_12_	**428.55**	439.24	**11.609**	**16.598**
SARIMA(0,1,1)(0,1,1)_12_^*^	429.05	**437.06**	11.668	17.248
SARIMA(0,1,1)(2,1,0)_12_	429.24	439.93	11.723	16.766
SARIMA(1,1,1)(0,1,1)_12_	430.96	441.65	11.671	17.200
SARIMA(0,1,1)(2,0,0)_12_	459.25	470.37	11.333	18.603
ARIMA(1,1,1) (No Seasonal)	469.18	477.52	11.885	20.329

AIC, Akaike Information Criterion; BIC, Bayesian Information Criterion; RMSE, Root Mean Square Error; MAPE, Mean Absolute Percentage Error. ^∗^Indicates the optimal model selected based on the Principle of Parsimony (lowest BIC). Bold values indicate the best performance in each category.

### 3.4 Parameter estimation and diagnostic verification

The optimal *SARIMA*(0,1,1) × (0,1,1)_12_ model parameters were estimated using the Maximum Likelihood method (Table 2). Both the non-seasonal moving average coefficient (*MA*1 = −0.7038) and the seasonal moving average coefficient (*SMA*1 = −0.6620) were statistically significant (*p* < 0.001), indicating strong temporal dependencies in both the immediate past and the annual cycle. Diagnostic checks on the residuals confirmed the model’s adequacy. The residual time series plot (Supplementary Figure S3) exhibited no discernible patterns, and the autocorrelation function (ACF) of the residuals remained within the significance bounds, suggesting that the residuals constituted white noise. This finding was further corroborated by the Ljung-Box *Q* test (*p* > 0.05), which did not reject the null hypothesis of independence. Consequently, the model was deemed statistically robust and suitable for forecasting.

**Table 2:** Estimates and standard error of optimal SARIMA(0,1,1)(0,1,1)_12_ model parameters.

Component	Model term	Estimate	Standard_Error	*t*-value	*p*-value
Non-Seasonal	MA (1)	−0.7038	0.0722	−9.75	< 0.001
Seasonal	Seasonal MA (1)	−0.6620	0.0971	−6.82	< 0.001

### 3.5 Model benchmarking across methodological families

To rigorously evaluate the predictive performance of the proposed *SARIMA*(0,1,1) × (0,1,1)_12_ baseline, an empirical benchmarking analysis was conducted against a representative suite of alternative forecasting models using the 2024 validation dataset (Table 3).

**Table 3:** Forecasting performance benchmarking across representative model families (2024 testing set).

Model architecture	MAE	RMSE	MAPE (%)
*SARIMA*(0,1,1) × (0,1,1)_12_*	9.12	11.93	29.52
Standard *ARIMA*(1,1,1)	8.69	10.75	24.48
Holt-Winters Additive Exponential Smoothing	9.44	13.28	31.89
Random Forest Regression	9.66	13.51	33.58

^∗^Indicates the proposed seasonal baseline model. MAE, Mean Absolute Error (cases/month); RMSE, Root Mean Square Error; MAPE, Mean Absolute Percentage Error.

Overall, traditional statistical and smoothing approaches demonstrated superior predictive stability compared to machine learning baselines when evaluated on moderate monthly surveillance samples. Although the non-seasonal Standard *ARIMA* (1,1,1) yielded a marginally lower MAE (8.69 vs. 9.12 cases/month), the difference (< 0.43 cases/month) is clinically negligible in routine public health practice; more importantly, standard ARIMA lacks the seasonal parameters necessary to capture annual epidemic waves. Holt-Winters exponential smoothing effectively captured local variations but showed higher variance during rapid trend shifts. Random Forest Regression exhibited mild overfitting (MAE = 9.66, MAPE = 33.58%), reflecting the intrinsic challenges supervised machine learning algorithms face with small-to-moderate epidemiological time series. Consequently, the parsimonious *SARIMA*(0,1,1) × (0,1,1)_12_ architecture provides the optimal balance between predictive precision, structural parsimony, and explicit epidemiological interpretability. Full comparative evaluations encompassing all six evaluated architectures (including Facebook Prophet and XGBoost) are provided in Supplementary Table S4.

### 3.6 Multi-year rolling window cross-validation

To evaluate model stability across changing temporal contexts and mitigate single-year evaluation bias, a 3-year expanding rolling window validation scheme was conducted for the proposed SARIMA model across 2022, 2023, and 2024 (Table 4).

**Table 4:** Multi-year rolling window forecasting performance for the proposed SARIMA model.

Evaluation year	Training period	Testing period	MAE	RMSE	MAPE (%)
2022	2014.01–2021.12	2022.01–2022.12	13.88	16.36	49.03
2023	2014.01–2022.12	2023.01–2023.12	11.71	14.47	24.52
2024	2014.01–2023.12	2024.01–2024.12	9.12	11.93	29.52
Average	-	-	11.57	14.26	34.36

Testing horizons consist of out-of-sample 12-month forecasts for each evaluation year. MAE, Mean Absolute Error (cases/month); RMSE, Root Mean Square Error; MAPE, Mean Absolute Percentage Error.

The multi-year rolling validation demonstrated remarkable temporal generalizability and performance stability. The annual MAE steadily improved from 13.88 in 2022 to 11.71 in 2023, and further to 9.12 in 2024, yielding a multi-year average MAE of 11.57 cases/month, an average RMSE of 14.26, and a MAPE of 34.36%. This continuous error reduction highlights the model’s capacity to adapt to longitudinal data trends without suffering from parameter degradation.

### 3.7 Out-of-sample forecasting and the “Spring Festival Effect”

The model’s performance was validated using actual surveillance data from January to December 2024 (Figure 4). Overall, the model exhibited high accuracy in predicting the total annual disease burden, with a relative error of merely 6.17% (Forecasted: 499 vs. Reported: 470) (Table 5). However, a detailed monthly analysis uncovered a notable behavioral irregularity. The model notably overestimated the incidence in January and February, with actual reported cases falling substantially below the lower bounds of the 95% PIs (Table 5). This deviation beyond the uncertainty intervals closely aligns with the “Spring Festival Effect,” during which healthcare-seeking behavior tends to decrease over the Lunar New Year holidays. Significantly, this was succeeded by a marked “catch-up” phenomenon in March, with the actual reported cases spiking (n = 57), closely mirroring the model’s projection (Relative Error = −7.02%). Although the point forecast for November (22 cases) was lower than the actual reported number (40 cases), leading to a higher relative error, the observed value still resided comfortably within the model’s 95% prediction interval (6–49 cases). This suggests that, apart from the systematic bias noted during the Spring Festival, the model continues to demonstrate robustness in accommodating random fluctuations for the remainder of the year.

**Figure 4:**
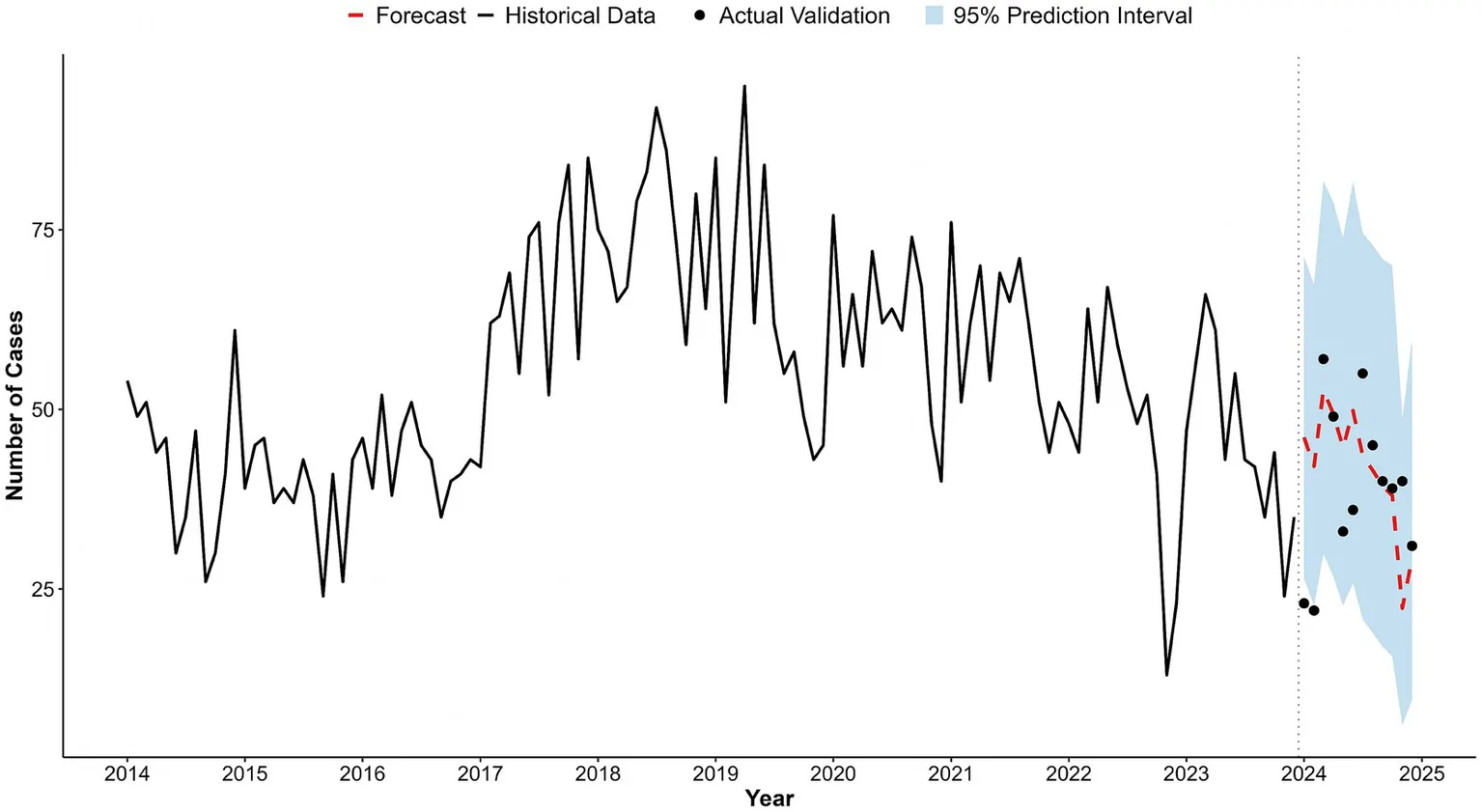
SARIMA(0,1,1)(0,1,1)_12_ model verification and forecasting of pulmonary tuberculosis cases in Jiulongpo District, Chongqing, 2024 (the shaded band indicates the 95% prediction intervals).

**Table 5:** Comparison of reported and forecasted pulmonary tuberculosis cases in Jiulongpo District, Chongqing, 2024.

Month	Actual cases	Forecasted cases (95% PI)	Relative error (%)
Jan	23	46 (26–71)	100.00
Feb	22	42 (23–67)	90.91
Mar	57	53 (30–82)	−7.02
Apr	49	49 (27–79)	0.00
May	33	45 (23–74)	36.36
Jun	36	50 (26–82)	38.89
Jul	55	44 (21–75)	−20.00
Aug	45	42 (19–73)	−6.67
Sep	40	39 (17–71)	−2.50
Oct	39	38 (16–70)	−2.56
Nov	40	22 (6–49)	−45.00
Dec	31	29 (10–60)	−6.45
Total	470	499	6.17

## 4 Discussion

This study presents a thorough 11-year longitudinal examination of PTB epidemiology in Jiulongpo District. The consistent statistically significant decrease observed from 2014 to 2023 corresponds with national and global epidemiological patterns (3, 27, 28), indicating the enduring efficacy of the DOTS strategy and contemporary control measures implemented during China’s recent Five-Year Plans (29, 30). This decreasing trend reflects similar observations in other regions of China, such as Sichuan (31) and Zhejiang (32), albeit with varying decline rates attributable to regional socioeconomic distinctions. A critical insight from our analysis is the profound impact of policy shifts on surveillance continuity. The structural break identified in 2017–2018 underscores a common paradox in global public health: upgrading diagnostic criteria—such as the WS 196–2017 standard which newly mandated the inclusion of tuberculous pleurisy (12, 33)—improves overall case ascertainment but artificially disrupts the historical baseline. It is crucial to interpret this upsurge not as a failure in disease management, but as a beneficial outcome of heightened surveillance sensitivity that uncovered a backlog of misclassified cases. However, if left unadjusted, such policy-induced artifacts can lead to gross misinterpretations of epidemiological trends, potentially masking the true efficacy of control programs like the DOTS strategy. By retrospectively standardizing this data discontinuity, our study demonstrates a vital methodological step: epidemiological harmonization is an important prerequisite before utilizing machine learning or time-series models to evaluate long-term public health interventions. Furthermore, because the 2018 policy change was a statistical reclassification rather than a genuine epidemiological shift, retrospective harmonization followed by conventional SARIMA modeling was preferred over intervention-based approaches. This allowed us to reconstruct a consistent historical baseline for operational forecasting rather than evaluating causal impact.

Accurate forecasting is essential for the proactive allocation of public health resources. Recent studies, such as the comprehensive work by Baker et al., have clearly demonstrated the immense value of predictive modeling for supporting public health surveillance and optimizing resource allocation during infectious disease outbreaks (10). Although standard ARIMA models are commonly employed, they frequently fail to account for the intricate periodicity associated with infectious diseases (8). Our findings indicate that the SARIMA(0,1,1) × (0,1,1)_12_ model provides a superior fit for PTB surveillance data in this region, aligning with previous research on the effectiveness of SARIMA in seasonal disease forecasting (34, 35) and TB tracking across various contexts, including Algeria (36), Iran (37), and other provinces in China (38). While the SARIMA framework is well-established, we explicitly note that the primary contribution of this study lies in the epidemiological standardization and systematic interpretation of reporting delays, rather than algorithmic innovation. A significant methodological insight from this study is the deliberate selection of a parsimonious model over more complex alternatives, which is now strongly justified through our rigorous empirical benchmarking. Guided by the Principle of Parsimony (Occam’s Razor) (39), our comparative analysis demonstrated that advanced machine learning algorithms (e.g., Random Forest) exhibited tendencies to overfit the noisy surveillance data. However, as these baselines were implemented without exhaustive hyperparameter tuning, the observed performance differences may partially reflect implementation choices rather than inherent methodological limitations. Conversely, although non-seasonal standard ARIMA smoothed out fluctuations effectively, it completely fails to explicitly isolate biological seasonal parameters. Thus, the selected SARIMA framework provides an appropriate balance between predictive stability and epidemiological interpretability within the present experimental setting. In real-world epidemiological forecasting, surveillance data are inherently noisy due to reporting lags and behavioral fluctuations. By prioritizing the Bayesian Information Criterion (BIC), we selected the simpler “Airline Model,” effectively mitigating the risk of overfitting to this noise. Furthermore, the robustness of this selected baseline was rigorously confirmed through a multi-year rolling window cross-validation (2022–2024). By iteratively expanding the evaluation horizon, we demonstrated that the model’s accuracy is a temporally stable performance rather than a coincidental artifact of a single testing split. The residuals of the model were confirmed to be white noise, and its high accuracy in predicting the total annual caseload for 2024 (Relative Error: 6.17%) substantiates its robustness as a reliable tool for estimating the district’s true annual disease burden.

The substantial divergence between our forecasted “biological baseline” and the actual reported cases in early 2024 provides valuable quantitative insights into the influence of festival holidays on disease surveillance. Specifically, the observed values fell significantly below the model’s 95% prediction intervals in January and February. However, this discrepancy is not a predictive failure; rather, it effectively highlights anomalies consistent with the “Spring Festival Effect.” This phenomenon extends beyond a mere statistical anomaly; it reflects a profound socio-cultural disruption where massive holiday-associated population migration, combined with a traditional reluctance to seek medical care during the Lunar New Year, likely contributes to a temporary “diagnostic filter” (15). It is also plausible that this deviation encompasses temporary operational disruptions and reduced staffing in local healthcare facilities during the holiday period (40). Similar holiday-associated reporting delays have been universally documented across various cultures and notifiable infectious diseases (41, 42), emphasizing that behavioral seasonality can temporarily override biological transmission dynamics. Conversely, the model accurately predicted the increase in March. This subsequent “catch-up” phenomenon—where reported cases surpassed the forecast, effectively bridging the earlier gap—confirms that the winter shortfall represented a backlog of delayed diagnoses rather than absent cases. This pattern is further elucidated by the biological seasonality of tuberculosis, which typically induces a spring peak due to factors such as Vitamin D deficiency during winter and increased indoor crowding (43, 44). Consequently, the model serves as both an anomaly detection tool and a quantitative reference standard: the disparity between the forecasted baseline and the reported data during winter is consistent with temporary reporting disruptions, rather than providing definitive measurements of delayed notifications. For health authorities, this analysis serves as a caution against misinterpreting low winter reporting rates as a genuine decline in transmission. More importantly, this framework translates statistical residuals into actionable public health intelligence. Instead of a uniform resource allocation approach, local CDCs can utilize the calculated winter shortfall to dictate the exact scale of Active Case Finding (ACF) and targeted contact tracing required in March. Allocating resources in anticipation of this inevitable post-holiday surge is critical to intercepting delayed transmission chains.

Several limitations must be acknowledged. First, this study relies on surveillance data from a single geographical area, Jiulongpo District in Chongqing. Given the vast regional disparities in socioeconomic status, population mobility, and healthcare access across China, the external validity of our specific model parameters is inherently limited. The current results may not seamlessly apply elsewhere, and this framework must be strongly emphasized as requiring recalibration with localized data before broader geographic extrapolation. Nevertheless, this methodological workflow—integrating epidemiological standardization with benchmarked forecasting—holds broad public health significance. While the overarching workflow—integrating data standardization with benchmarked forecasting—is broadly transferable, its successful adaptation to monitor other infectious diseases globally strictly depends on the availability of disease-specific retrospective classification data and a clear understanding of local surveillance definitions. Second, the SARIMA model is univariate and relies exclusively on historical data, failing to incorporate external covariates such as meteorological factors or economic indicators. Third, although our retrospective harmonization mitigated the primary reporting discontinuity caused by the 2018 classification change, it cannot completely eliminate unmeasured “diagnostic criteria drift.” Gradual changes, such as improved clinician awareness, advancements in diagnostic technologies, and enhanced reporting completeness following the policy shift, may still introduce residual surveillance bias into the long-term trend. Furthermore, the model is unable to predict sudden external shocks (e.g., future pandemics) that may disrupt surveillance systems. Fourth, as detailed in the methodology, a major public health jurisdiction transfer in early 2025 structurally altered the local population denominator. Consequently, our forecasting framework is currently constrained to the 2014–2024 closed historical cohort. Future research will require establishing a newly calibrated demographic baseline for the restructured administrative areas. Finally, the “Spring Festival Effect” introduces a moving holiday bias, as the date of the Lunar New Year varies annually. Recent advancements, such as the collaborative machine learning framework for disease prediction proposed by Baker et al., highlight the tremendous potential of integrating artificial intelligence with conventional epidemiological approaches (11). Discussing these broader contexts strongly justifies the future adoption of hybrid forecasting models. Future studies could investigate advanced machine learning techniques or hybrid models, such as the SARIMA-NNNAR (45), SARIMA-NARX (46), and SARIMA-NARNNX hybrid models (47), to more effectively capture these non-linear complexities and enhance short-term prediction accuracy.

## 5 Conclusion

This study establishes a practical framework for infectious disease forecasting, specifically addressing surveillance disruptions associated with health policy shifts and festival holidays. First, it demonstrates that rigorous epidemiological standardization effectively mitigates surveillance artifacts, ensuring the longitudinal utility of historical data despite policy-induced structural breaks. Second, extensive benchmarking and multi-year rolling cross-validation confirm that the parsimonious SARIMA model provides robust temporal stability, successfully avoiding the overfitting tendencies observed in complex machine learning alternatives. By applying this validated baseline, we move beyond basic incidence prediction to practical anomaly detection. By quantitatively evaluating the “Spring Festival Effect,” the framework helps differentiate socio-cultural reporting delays from natural biological seasonality, revealing patterns consistent with a substantial backlog of delayed notifications. Consequently, public health authorities could transition from uniform resource allocation to a dynamic, data-driven strategy. Although the specific model parameters require localized recalibration before broader geographic application, proactively deploying Active Case Finding (ACF) and targeted screening in the immediate post-holiday period remains a critical strategy to manage these reporting backlogs.

## Data Availability

The original contributions presented in the study are included in the article/Supplementary material, further inquiries can be directed to the corresponding authors.
