# Supplementary material for: Impact of policy shifts and festival holidays on tuberculosis surveillance: a time-series analysis in Southwest China (2014–2024)
Source: Front Public Health. 2026 Sep 23;14:1900071. doi: 10.3389/fpubh.2026.1900071 (PMC13642786; doi:10.3389/fpubh.2026.1900071)
Supplement: Supplementary file 1 [file Data_Sheet_1.pdf]

Supplementary Table S1. Distribution of reported pulmonary tuberculosis cases by sex, age, and occupation in Jiulongpo District, Chongqing, 2014–2023.

Variable	2014 n=514	2015 n=458	2016 n=520	2017 n=795	2018 n=895	2019 n=762	2020 n=743	2021 n=725	2022 n=563	2023 n=552	Total N=6527	%
Sex												
Male	341	294	373	557	614	493	488	500	356	384	4400	67.41
Female	173	164	147	238	281	269	255	225	207	168	2127	32.59
Age. years												
<15	2	4	7	8	9	4	6	5	5	3	53	0.81
15-24	98	63	90	112	127	108	91	93	63	49	894	13.70
25-34	94	109	101	146	158	175	179	135	96	85	1278	19.58
35-44	79	52	60	81	97	85	71	75	67	52	719	11.02
45-54	94	84	83	169	153	126	124	139	90	80	1142	17.50
55-64	80	63	86	133	179	120	127	131	106	103	1128	17.28
≥65	67	83	93	146	172	144	145	147	136	180	1313	20.12
Occupation												
Workers	54	50	122	150	77	57	54	48	24	24	660	10.11
Teachers and students	30	22	37	49	68	59	43	52	30	23	413	6.33
Farmers	57	61	75	107	107	113	138	156	107	94	1015	15.55
Retired personnel	73	84	102	116	123	97	93	74	75	81	918	14.06
Housework and unemployment	210	182	107	254	363	316	288	262	239	264	2485	38.07
Others	90	59	77	119	157	120	127	133	88	66	1036	15.87

Supplementary Table S2. Top regions with the highest number of reported pulmonary tuberculosis cases in Jiulongpo District, Chongqing, 2014–2023.

Year	Rank 1 (n)	Rank 2 (n)	Rank 3 (n)	Rank 4 (n)	Rank 5 (n)
2014	Yangjiaping (107)	Shiqiaopu (59)	Shipingqiao (53)	Huangjueping (42)	Zhongliangshan (38)
2015	Yangjiaping (99)	Shiqiaopu (56)	Jiulong (44)	Xiejiawan (43)	Zhongliangshan (41)
2016	Yangjiaping (113)	Shiqiaopu (65)	Shipingqiao (48)	Jiulong (41)	Huangjueping* (38)
2017	Yangjiaping (157)	Shiqiaopu (106)	Shipingqiao (72)	Jiulong (70)	Huayan Town (64)
2018	Yangjiaping (170)	Shiqiaopu (93)	Huayan Town (89)	Shipingqiao (82)	Xiejiawan (62)
2019	Yangjiaping (132)	Shiqiaopu (102)	Huayan Town (73)	Shipingqiao (60)	Jiulong (53)
2020	Yangjiaping (115)	Shiqiaopu (92)	Shipingqiao (80)	Xiejiawan (60)	Jiulong (57)
2021	Yangjiaping (93)	Shiqiaopu (82)	Shipingqiao (74)	Xiejiawan (65)	Jiulong (62)
2022	Yangjiaping (76)	Shiqiaopu (64)	Xiejiawan (58)	Huayan Town(57)	Shipingqiao (48)
2023	Yangjiaping (72)	Shiqiaopu (63)	Xiejiawan (49)	Huayan Town (48)	Shipingqiao (42)
**Total**	**Yangjiaping (1134)**	**Shiqiaopu (779)**	**Shipingqiao (597)**	**Huayan Town (521)**	**Xiejiawan (513)**

Note: Regions without a specific suffix (e.g., Town) refer to Subdistricts. * In 2016, Xiejiawan Subdistrict also reported 38 cases, tying for the 5th rank.

Supplementary Table S3. Annual population, number of cases, and incidence rates of pulmonary tuberculosis in Jiulongpo District, Chongqing, 2014–2023.

Year	Population	Cases (n)	Incidence rate (per 100,000)	χ^2_{trend}	*P*-value
2014	1,099,684	514	46.74		
2015	1,107,539	458	41.35		
2016	1,186,900	520	43.81		
2017	1,199,443	795	66.28		
2018	1,215,101	895	73.66		
2019	1,225,001	762	62.20	-5.642	<0.001
2020	1,233,011	743	60.26		
2021	1,526,823	725	47.48		
2022	1,540,221	563	36.55		
2023	1,535,963	552	35.94		

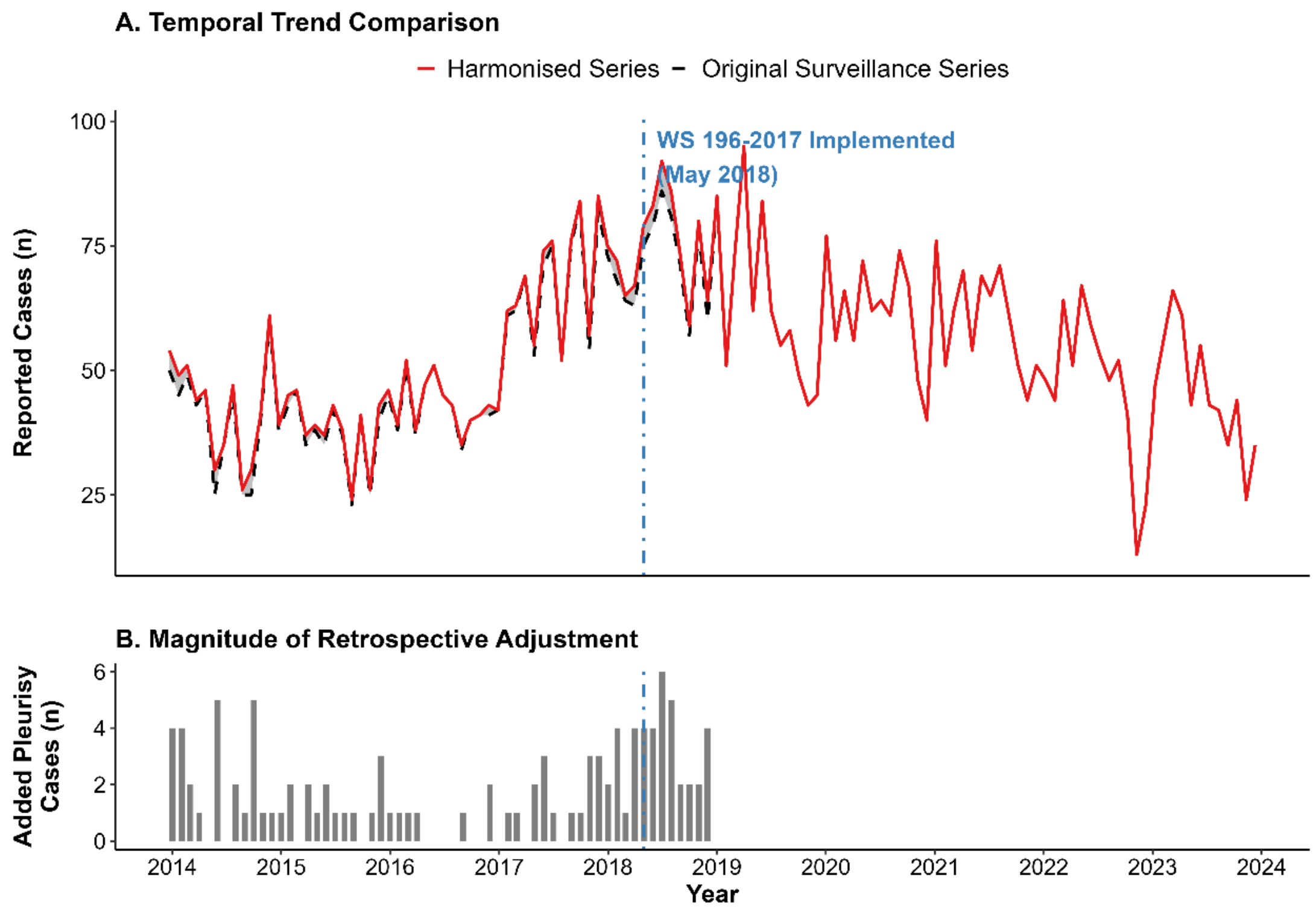

Supplementary Figure S1. Empirical validation of the retrospective data harmonisation procedure. (A) Direct comparison between the original unadjusted surveillance series (black dashed line) and the harmonised series (red solid line). (B) The exact magnitude of the retrospective adjustment, representing the monthly aggregated tuberculous pleurisy cases prior to the nationwide implementation of the WS 196-2017 standard in May 2018.

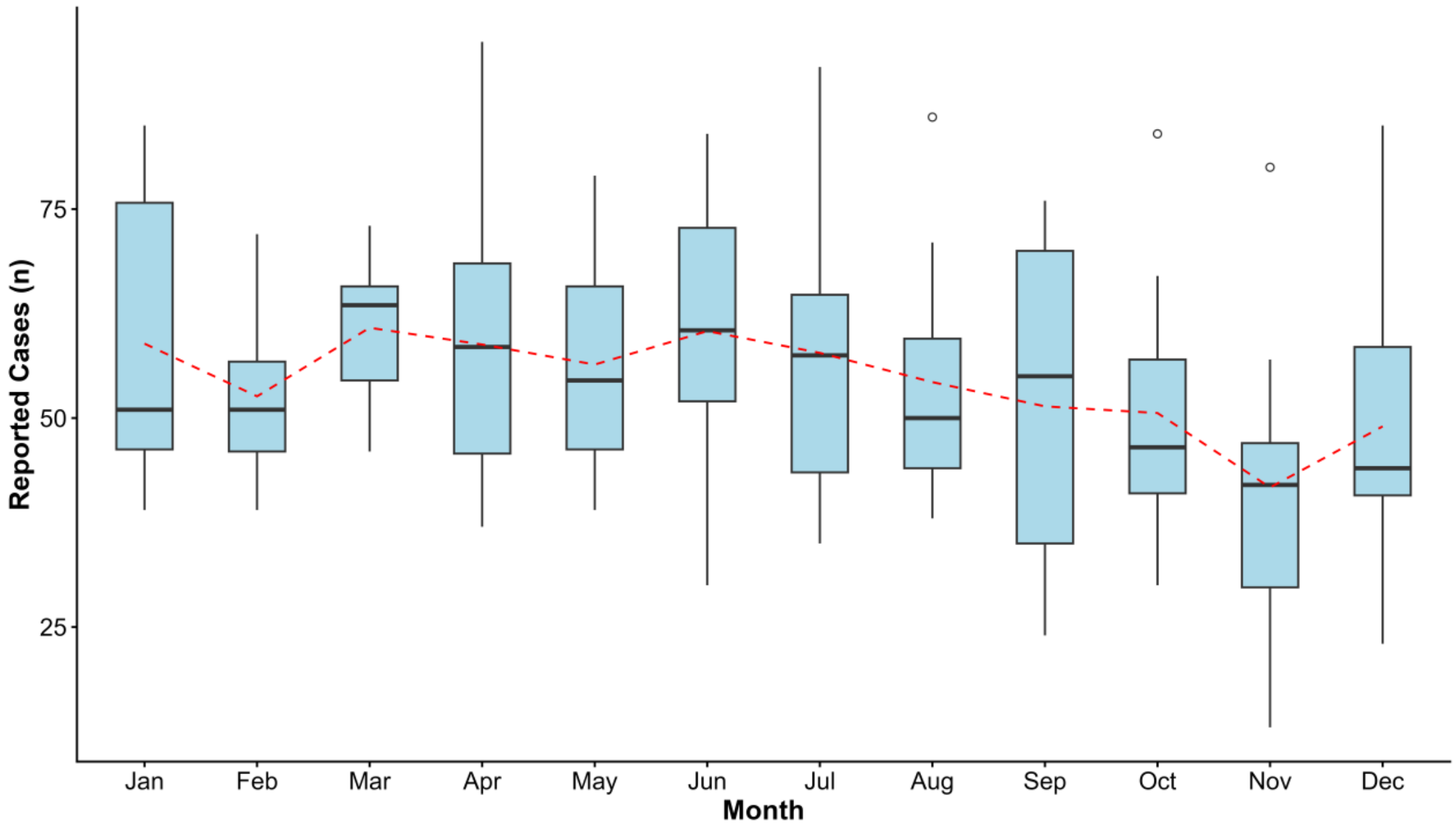

Supplementary Figure S2. Monthly incidence cases of pulmonary tuberculosis in Jiulongpo District, Chongqing, 2014–2023.

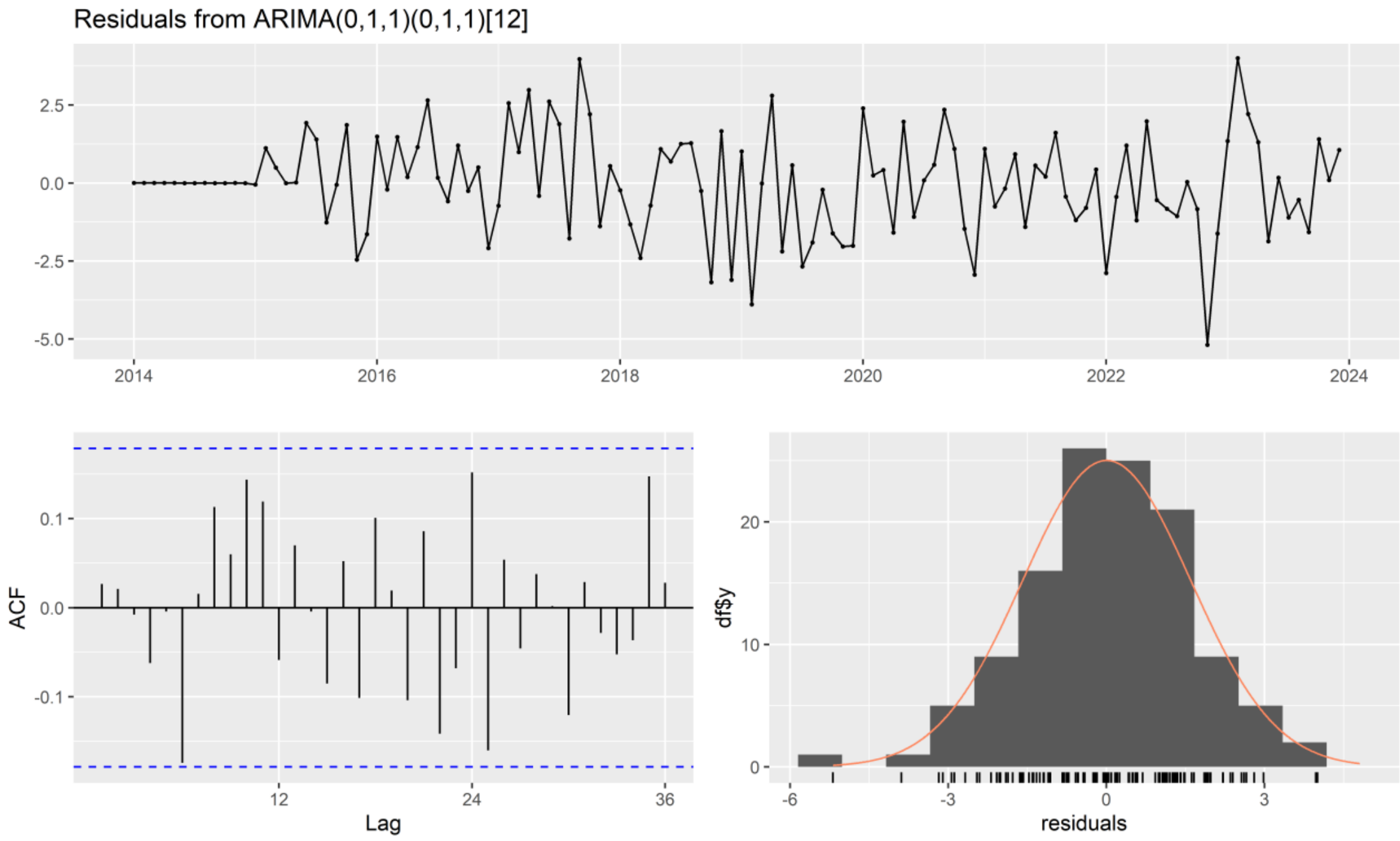

Supplementary Figure S3. Residuals from SARIMA$(0,1,1)(0,1,1)_{12}$.

Supplementary Table S4. Forecasting performance benchmarking across representative model families (2024 testing set)

Model architecture	MAE	RMSE	MAPE (%)
SARIMA$(0,1,1)\times(0,1,1)_{12}$	9.12	11.93	29.52
Standard *ARIMA*(1,1,1)	8.69	10.75	24.48
Facebook Prophet	9.26	12.71	31.74
Holt-Winters Additive Exponential Smoothing	9.44	13.28	31.89
Random Forest Regression	9.66	13.51	33.58
XGBoost Regression	12.37	15.73	40.53

Note: * Indicates the proposed seasonal baseline model. MAE: Mean Absolute Error (cases/month); RMSE: Root Mean Square Error; MAPE: Mean Absolute Percentage Error.

Supplementary Text S1. Methodological Implementation and Parameter Settings for Benchmark Models

To ensure full transparency and reproducibility of the comparative analysis presented in Section 3.5, the detailed software implementations, feature engineering procedures, and hyperparameter settings for all benchmarked models are described below.

1. Software Environment

All models were implemented using R software (version 4.3.1). The primary packages utilized included forecast (v8.21.1) for classical statistical models, prophet (v1.0) for Facebook Prophet, randomForest (v4.7-1.1) for Random Forest regression, and xgboost (v1.7.5.1) for Extreme Gradient Boosting.

2. Feature Engineering for Machine Learning Models

Unlike traditional time-series models that natively handle temporal dependencies, the machine learning models (Random Forest and XGBoost) required explicit feature engineering. The target variable (y) was the monthly reported TB cases. To capture the autoregressive and seasonal dynamics, we engineered the following features:

- Autoregressive Lags: 12 distinct lag features (Lag_1 to Lag_{12}) representing the historical incidence of the preceding 12 months. (Missing values generated by shifting were omitted, resulting in a training set spanning from January 2015 to December 2023).
- Temporal Features: A continuous Time_Index (1 to 120) to capture long-term linear trends, and a categorical Month variable (1 to 12) to capture inherent monthly seasonality.
- Recursive Forecasting: For the 12-month out-of-sample forecasts (January to December 2024), a recursive 1-step-ahead forecasting strategy was applied. The predicted value for month t was iteratively appended to the historical sequence to construct the necessary lag features for forecasting month $t + 1$.

3. Model Specifications and Hyperparameter Settings

To evaluate the baseline performance of each methodological family without introducing bias from exhaustive, resource-intensive global optimization, standard structural configurations and default hyperparameters were employed.

- Proposed SARIMA: Implemented via the Arima() function. Order = (0,1,1), Seasonal Order = (0,1,1), Period = 12.
- Standard ARIMA: Implemented via the Arima() function. Order = (1,1,1) with no seasonal components.
- Holt-Winters Exponential Smoothing: Implemented via the HoltWinters() function with an "additive" seasonal component.
- Facebook Prophet: Implemented via the prophet() function. yearly.seasonality = TRUE, weekly.seasonality = FALSE, daily.seasonality = FALSE. Other settings (e.g., changepoint prior scale) were kept at package defaults.
- Random Forest Regression: Implemented via the randomForest() function. Number of trees (ntree) = 500. The number of variables randomly sampled as candidates at each split (mtry) was left at the default setting for regression (one-third of the total features). A fixed random seed (123) was used for reproducibility.
- XGBoost Regression: Implemented via the xgboost() function. Number of boosting rounds (nrounds) = 100; Objective function = reg:squarederror. Learning rate (η) and maximum tree depth (max_depth) were retained at default values (0.3 and 6, respectively). A fixed random seed (123) was used for reproducibility.

4. Notes on Hyperparameter Optimization

It is important to acknowledge that the machine learning models evaluated in this study did not undergo comprehensive hyperparameter optimization (e.g., extensive grid search or randomized search). Consequently, the observed tendency of the Random Forest and XGBoost models to overfit the historical data may partially reflect these specific implementation choices rather than an inherent, universal limitation of machine learning methodologies. Future studies utilizing rigorously tuned architectures could potentially yield different performance dynamics.
